# Land-Use Driven Changes in Soil Microbial Community Composition and Soil Fertility in the Dry-Hot Valley Region of Southwestern China

**DOI:** 10.3390/microorganisms10050956

**Published:** 2022-05-02

**Authors:** Taicong Liu, Zhe Chen, Li Rong, Xingwu Duan

**Affiliations:** 1 Institute of International Rivers and Eco-Security, Yunnan University, Kunming 650500, China; taicongliu@163.com; 2 Yunnan Key Laboratory of Plant Reproductive Adaptation and Evolutionary Ecology, Yunnan University, Kunming 650091, China; zhechen2019@ynu.edu.cn; 3 Yunnan Key Laboratory of International Rivers and Trans-Boundary Eco-Security, Yunnan University, Kunming 650091, China

**Keywords:** maize land, sugarcane land, forest land, barren land, bacteria and fungi

## Abstract

The Dry-Hot Valley is a unique geographical region in southwestern China, where steep-slope cultivation and accelerating changes in land-use have resulted in land degradation and have aggravated soil erosion, with profound impacts on soil fertility. Soil microbes play a key role in soil fertility, but the impact of land-use changes on soil microbes in the Dry-Hot Valley is not well known. Here, we compared characteristics and drivers of soil microbial community composition and soil fertility in typical Dry-Hot Valley land uses of sugarcane land (SL), forest land (FL), barren land (BL) converted from former maize land (ML), and ML control. Our results showed that BL and SL had reduced soil organic carbon (SOC), total nitrogen (TN), and total potassium (TK) compared to ML and FL. This indicated that conversion of ML to SL and abandonment of ML had the potential to decrease soil fertility. We also found that fungal phyla *Zoopagomycota* and *Blastocladiomycota* were absent in SL and BL, respectively, indicating that land-use change from ML to SL decreased the diversity of the bacterial community. Redundancy analysis indicated that the relative abundance of bacterial phyla was positively correlated with TN, SOC, and available potassium (AK) content, and that fungal phyla were positively correlated with AK. Land-use indirectly affected the relative abundance of bacterial phyla through effects on soil moisture, clay, and AK contents, and that of fungal phyla through effects on clay and AK contents. In addition, land-use effects on bacteria were greater than those on fungi, indicating that bacterial communities were more sensitive to land-use changes. Management regimes that incorporate soil carbon conservation, potassium addition, and judicious irrigation are expected to benefit the stability of the plant–soil system in the Dry-Hot Valley.

## 1. Introduction

Land-use is the main driver of environmental changes due to modifications of the physical, chemical, and biological properties of soil [1]. For example, land-use changes significantly affect soil organic carbon (SOC), which plays a crucial role in many soil functions and ecosystem services [1,2,3]. SOC tends to decrease when forests are converted to croplands due to the loss of tree biomass carbon [4]. In contrast, croplands converted to forests usually increase SOC [5]. Unsustainable land-use changes can lead to soil carbon loss and soil degradation [6], trigger soil erosion [7], decrease soil productivity, and threaten food security [8,9]. With the growing human population and food demands [8], there is an urgent need to practice sustainable conversion of land to farmland.

Microbiota in soil ecosystems are highly abundant, diverse, and active [10]; they are the drivers of soil-organic-matter processes and play a key role in SOC stabilization [11]. Changes in microbial community composition in response to ecosystem disturbances may result in large net changes in SOC, with implications for soil productivity and fertility [12,13]. Hence, an understanding of the changing characteristics and drivers of soil microbial community composition under different land uses is vital to regulating SOC stabilization and improving soil fertility. Many studies have shown that land-use can change the composition and diversity of soil microbial communities [14,15,16]. Other studies, however, indicated that land-use had no effect on soil microbial community composition [17,18,19]. Usually, land-use determines the quantity and quality of aboveground and belowground litter [20], which affects soil organic carbon [21] and soil nutrients [22]. Litterfall comprises the resources for the soil food web, shaping the soil microbial community [23]. On the other hand, variability in other key soil properties, such as soil physicochemical composition, which is influenced by land-use-associated vegetation, can result in diversity in microbial communities [24,25,26]. 

The Dry-Hot Valley region is located mainly in southwestern China. It is characterized by steep slopes and high temperature and evaporation [27]. Due to plenty of light and heat, this area is used extensively for the production of maize, tropical crops, and fruits. However, steep slopes combined with concentrated rainfall have resulted in severe soil erosion [28] and loss of soil fertility [29], leading to the abandonment of farmland [30]. Other farmland on sleep slopes has been converted into forest land under the policy of returning farmland to forest. Additionally, the low profitability of native crops and a reduction in rural labor due to out-migration have promoted the cultivation of crops with high economic value, such as sugarcane [31]. Consequently, the soil ecosystem in the Dry-Hot Valley has become more fragile [32]. A number of reports have focused on soil erosion and ecological restoration in dry-hot valleys [33,34,35]. However, it is not clear whether land-use changes affect soil microbial community composition, and if so, how. In this study, we focused on soil microbial community composition and soil fertility in different land uses in the Dry-Hot Valley. Our objectives were to (1) determine the response to land-use changes of soil microbial communities and soil organic carbon, total nitrogen, phosphorus, and other abiotic soil factors, and (2) identify the relationship between the microbial community and abiotic factors within land-use types. We hypothesized that: (1) the contents of SOC and TN would be lower in SL and BL than in FL and ML, and that (2) land-use changes would induce changes in soil moisture, influencing soil microbial community composition.

## 2. Materials and Methods

### 2.1. Study Site

The experiment was conducted at the Yuanjiang Dry-Hot Valley State Field Station for Soil and Water Conservation Research (23°58′5″ N, 101°38′55″ E) in Yunnan Province, southwestern China. The site is at 542 m elevation with an average slope gradient of 27°. The annual average temperature and precipitation are approximately 23.9 °C and 781 mm, respectively. More than 80% of the precipitation occurs during the rainy season from late May to mid-October [30]. The annual mean potential evaporation is 2892 mm, resulting in extreme aridity. The soil type is classified as dry red soil (torrid red soil) in Chinese Soil Taxonomy, and Hapli-Ustic Ferrosol in the FAO soil classification system [36]. Maize (*Zea mays* L.) land, sugarcane (*Saccharum sinense* Roxb.) land, and forest land converted from farmland are the most common land-use types in the Dry-Hot Valley. 

**Figure 1:**
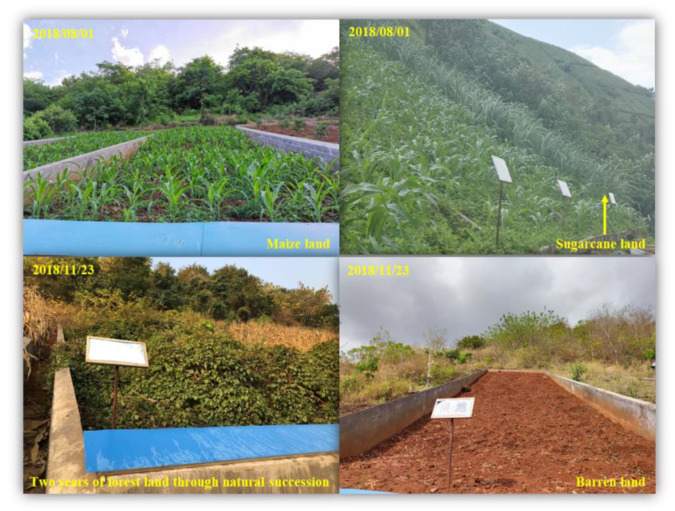
The experimental plots of four land-use types.

Sugarcane land (SL), forestland (FL) (also referred to as forestland converted from farmland), and barren land (BL) were established on a former maize field in a randomized design with four replicates and a plot size of 100 m^2^ (Figure 1, 20 m × 5 m in size, established on 11 May 2016). The left plots continued to grow maize and were defined as maize land (ML). Vegetation in the FL was allowed to regrow naturally through natural succession. A vegetation quadrat survey (20 m× 5 m) in August 2018 showed that the dominant woody plants in FL were *Albizia durazz*, *Bombax ceiba*, and *Broussonetia papyrifera*, and the dominant herbs were *Malva sinensis*, *Chromolaena odorata*, and *Vitaceae juss*. ML and SL were established using the local planting system. Maize was planted in early May, and fields were left fallow after harvest from October to January of the following year. Sugarcane was planted from December to February of the following year. In ML and SL, contour ridge tillage and uniform management practices were used without the application of fertilizer, and weed removal was performed by hand. BL represented abandoned farmland. In the Dry-Hot Valley, farmland is abandoned when extensive ephemeral erosion gullies develop [30]. These farmlands were commonly barren at the time of abandonment. For the purpose of this study, BL was kept barren, with weed removal during the experimental period to discourage regrowth to FL. 

### 2.2. Soil Sampling 

Soils were sampled on 23 November 2018. Five soil cores were taken from the upper soil layer (0–20 cm) of each plot using a soil auger (5 cm diameter); cores were mixed into one soil sample after removing visible debris and stones. A total of 16 samples were obtained from four types of land-use; the composite samples were divided into two parts. One part was placed on dry ice and stored in a freezer at −80 °C for 16S bacterial rRNA and ITS fungal gene analysis. The other part was passed through 2.0 mm and 0.149 mm sieves after air drying for determination of soil physical and chemical properties.

### 2.3. Analysis of Soil Physicochemical Properties

Soil pH was analyzed with a pH meter in a 1:2.5 ratio of soil to water (NY/T1377-2007). Soil moisture (SM) was measured gravimetrically after samples were oven-dried at 105 °C for 24 h [37]. Soil particle size distribution, including sand (2–0.02 mm), silt (0.02–0.002 mm), and clay (<0.002 mm) (United States Department of Agriculture, 1951), was analyzed using the International Pipette method with 10 mL of sodium hydroxide as a dispersant [38]. Soil samples were passed through a 0.149 mm mesh, treated with 15% phosphoric acid for 12 h to remove inorganic carbon, and air-dried [39]; soil organic carbon (SOC) was measured using an elemental analyzer (Flash 200 EA-HT, Thermo Fisher Scientific, Inc., Waltham, MA, USA). Total nitrogen (TN) was determined using the Kjeldahl method [40]. Total phosphorus (TP) and total potassium (TK) were measured using the molybdenum antimony colorimetric and flame photometer methods, respectively [41]. Available potassium (AK) was extracted with 2 mol L^−1^ HNO_3_(aq) and measured using flame atomic absorption spectrophotometry (Jitian, FIA6000, Beijing, China) [41]. Available phosphorus (AP) was extracted with 0.03 M NH_4_F-0.025 HCl(aq) and measured using a UV–vis spectrophotometer (Yuanxi, UV-5500, Shanghai, China) [41]. 

### 2.4. Total DNA Extraction, PCR Amplification, and Purification

Total DNA was extracted from all soil samples using a TIANamp Soil DNA Kit (Tiangen, Beijing, China) according to the manufacturer’s protocols, and DNA concentrations were measured with a UV–visible spectrophotometer (Thermo Scientific, Wilmington, DE, USA). The quality and quantity of DNA extracts were checked with electrophoresis (LIUYI Biotechnology Inc., Beijing, China) on a 1% agarose gel (MP Biomedicals, Cleveland, OH, USA). The DNA samples were diluted to a final concentration of 1 ng μL^−1^ using sterile water and stored at −80 °C until further use.

DNA samples were sent to Novogene Biological Information Technology Co. (Tianjin, China) for microbial community analysis. The hypervariable V4 regions of the bacterial 16S rRNA gene and hypervariable ITS1 regions of the fungal ITS gene were amplified using primers with specific barcodes. The bacterial-prime set was 515F (5′-GTGCCAGCMGCCGCGGTAA-3′) and 806R (5′-GGACTACHVGGGTWTCTAAT-3′) [42], and the fungal-prime set was ITS5-1737F (5′-GGAAGTAAAAGTCGTAACAAGG-3′) and ITS2-2043R (5′-GCTGCGTTCTTCATCGATGC-3) [43]. All PCR reactions were carried out in 30 μL reactions with 15 μL of Phusion^®^ High-Fidelity PCR Master Mix (New England Biolabs, Beverly, MA, USA); 0.2 μM of forward and reverse primers and about 10 ng template DNA were used. PCR was conducted using the following program: 3 min of denaturation at 98 °C, followed by 30 cycles of denaturation at 98 °C for 10 s, annealing at 50 °C for 30 s, elongation at 72 °C for 30 s, and a final extension at 72 °C for 5 min. 

Ten μL of PCR products was mixed with 1X loading buffer (containing SYBR green) and detected with electrophoresis on a 2% agarose gel. Samples with a band between 400–450 bp were selected for further analysis. PCR products were mixed in equidensity ratios, and the mixture was purified using the Gene JETTM Gel Extraction Kit (Thermo Scientific, Wilmington, DE, USA).

### 2.5. Library Preparation and Sequencing

Sequencing libraries were generated using the Ion Plus Fragment Library Kit 48 rxns (Thermo Scientific, Wilmington, DE, USA) following the manufacturer’s recommendations. Subsequently, index codes were added. Library quality was assessed on a Qubit 2.0 Fluorometer (Thermo Scientific, Wilmington, DE, USA) and Agilent Bioanalyzer 2100 system. After passing the test, the library was sequenced on an Ion S5^TM^ XL platform.

### 2.6. Data Preprocessing and Species Annotation

To obtain valid data for subsequent analysis, low-quality reads were cut, and sample data were split from the reads using Cutadapt v1.9.1 (e = 0.05, q = 17, m = 200 m, M = 400) [44]. Raw reads were obtained after preliminary quality control with barcode and primer sequences and processed to remove the chimera sequence. Sequences were compared with the species annotation database to detect the chimera sequence using VSEARCH v.2.14.1 [45]. Operational taxonomic unit (OTU) abundance information was normalized with a standard sequence number corresponding to the sample with the fewest sequences. 

Sequences with ≥97% similarity were assigned to the same OTUs for further annotation and cut off using UPARSE (Uparse v7.0.1001) [46]. The sequence with the highest frequency in OTUs was regarded as the representative sequence of OTUs according to the algorithm principle. Subsequently, taxonomic classification of each OTU-representative read was performed using the MOTHUR program via the Silva (SSU132) database, with a minimum identity of 80% [47].

### 2.7. Data Analysis

Venn diagrams in the vegan package of R software (version 4.0.2, University of Auckland, Auckland, New Zealand) were used to depict the number of unique and shared OTU numbers of bacteria and fungi for each sample in different land-use types. Shapiro–Wilk (S-W) and homogeneity tests were used to test data for normal distribution and homogeneity of variance. One-way analysis of variance (ANOVA) with Fisher’s least significant difference (LSD) test was used to determine differences in soil physicochemical properties and relative abundances of soil microbial phyla among land-use types. All analyses were conducted using SPSS 22.0 for Windows (SPSS Inc., Chicago, IL, USA). 

Beta-diversity analysis was performed using the unweighted UniFrac [48]. Moreover, non-metric multidimensional scaling (NMDS) was used to identify the relationships of microbial community composition in different land-use types using Bray–Curtis distances at the OTU level obtained from the vegan package of R software (version 4.0.2). Permutation multivariate analysis of variance (PERMANOVA) based on 999 permutations was used with Bray–Curtis distances and the Adonis function in the vegan package to assess significant differences in soil microbial community composition among land-use types. Analysis of similarities (ANOSIM) was used to determine whether significant differences existed in soil microbial community composition among land-use types based on the permutation test. Alpha diversity metrics (the Shannon–Wiener index) were calculated using the “diversity” function in the vegan package of R software (https://cran.r-project.org/bin/windows/base/old/4.0.2/, accessed on 24 November 2020).

Furthermore, redundancy analysis (RDA) was performed to visualize the relationships among soil physicochemical properties and the relative abundance of soil microbial phyla in different land-use types. RDA with forward selection was used to extract environmental variables that best explained variability in the relative abundance of soil microbial phyla. Variables with inflation factors > 10 were excluded when executing the summarized-effects procedure because of strong variability. RDA was performed using CANOCO 5.0 (http://www.canoco5.com/, accessed on 13 May 2020).

Structural equation models (SEM) were used in R software (version 4.0.2) to explore the relationships among land-use types, soil properties, and the relative abundance of soil microbial phyla [49]. First, an a priori model was established based on the known effects and relationships among environmental factors and land-use according to the RDA. Then, the model was parameterized using the dataset to test its overall goodness of fit as follows: (1) The χ^2^ test (χ^2^; model has a good fit when χ^2^ is low (~2) and *p* is high (traditionally >0.05)); (2) The root MSE of approximation (RMSEA; the model has a good fit when RMSEA is low (~0.05) and *p* is high (traditionally >0.05)); and (3) The standardized root mean square residual (SRMR) of the models (model has a good fit when SRMR < 0.08) [49]. 

## 3. Results

### 3.1. Soil Physicochemical Properties

**Figure 2:**
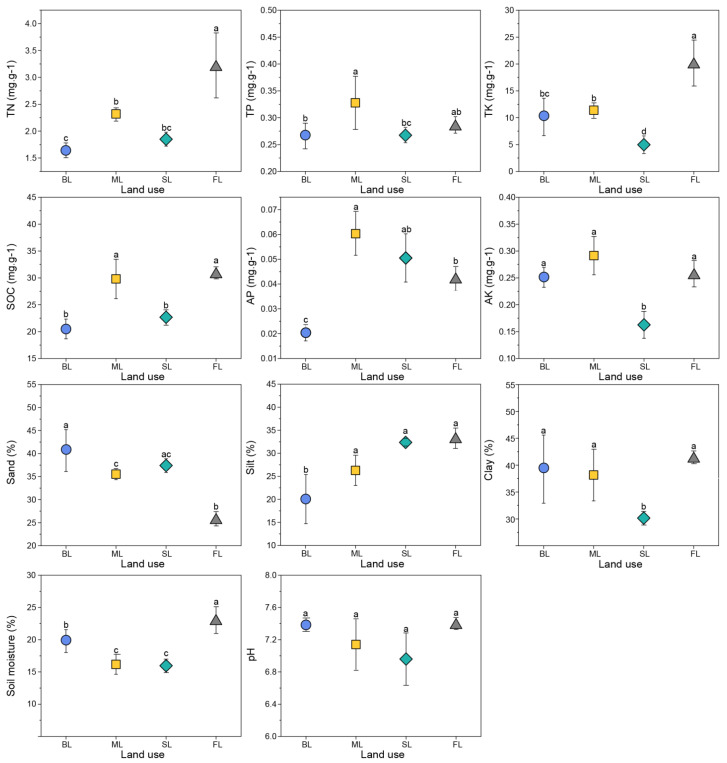
Soil physicochemical properties in four land-use types. Different letters denote significant differences (*p* < 0.05) between treatments as determined by quartic comparisons with Fisher’s least significant difference (LSD). Abbreviations: FL, forest land; SL, sugarcane land; ML, maize land; BL, barren land; TN, soil total nitrogen; TP, soil total phosphorus; TK, soil total potassium; SOC, soil organic carbon; AK, available potassium; AP, available phosphorus.

Soil properties were significantly influenced by land-use type (Figure 2, Supplementary Table S1). Contents of TN, TK, and soil moisture were significantly higher in land converted from ML to FL than in other land uses (*p* < 0.05). SOC was significantly lower in SL than in FL and ML (*p* < 0.05), indicating that the conversion from farmland to SL could induce a decline in soil fertility. Similarly, TK, AK, soil moisture, and clay contents were significantly lower in SL than in FL and ML (*p* < 0.05). Likewise, BL resulted in a loss of SOC and phosphorus (*p* < 0.01, Figure 2), with the AP content in BL soils being approximately one-third of that in ML soils. Soil pH was fairly consistent (ranging from 6.96 to 7.40) across all plots (Supplementary Table S1).

### 3.2. Analysis of Soil Microbial Sequences and OTUs

**Figure 3:**
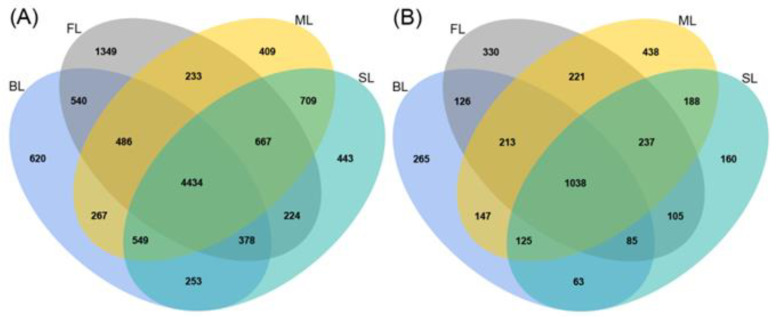
Venn diagrams showing the unique and shared OTUs of bacteria (**A**) and fungi (**B**) in four land-use types.

A total of 1,215,436 and 1,312,494 raw sequences were obtained for soil bacterial and fungal communities, respectively. The 16S rRNA sequences in each sample were normalized to 43,150, and the ITS sequences were rarefied to 59,938 for subsequent analysis (following analysis of rarefaction curves, Supplementary Figure S1). These sequences were identified as 11,780 bacterial OTUs and 4055 fungal OTUs. The unique and shared OTUs are shown in Figure 3. The number of shared OTUs among the four land-use types was 4434 for bacteria and 1038 for fungi. The four land-use types ranked FL > BL > SL > ML and ML > FL > BL > SL for the number of unique OTUs for bacteria and fungi, respectively (Figure 3).

### 3.3. Comparison of Soil Microbial Community Composition and Relative Abundance

**Figure 4:**
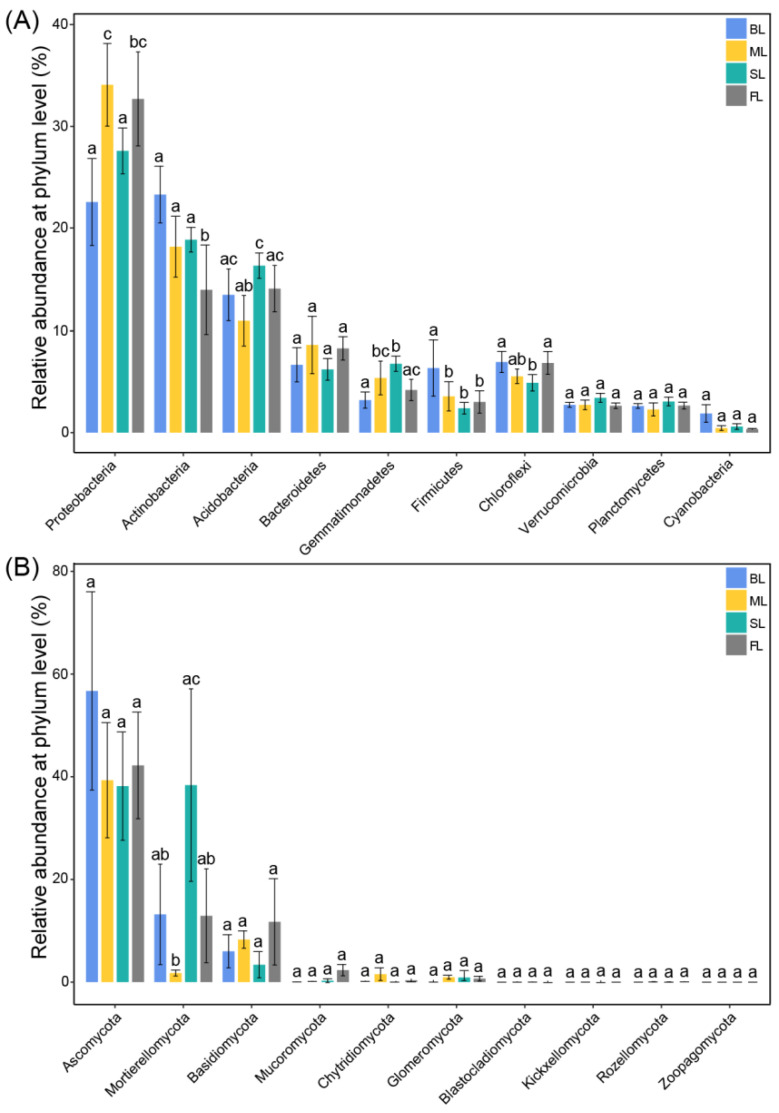
Relative abundance of the dominant bacteria (**A**) and fungi (**B**) in different land-use types. Error bars represent standard error (*n* = 4). Different letters above the bars indicate significant differences among different land-use types (*p* < 0.05) based on one-way ANOVA with the LSD test. Abbreviations: FL, forest land; SL, sugarcane land; ML, maize land; BL, barren land.

Sixty-nine phyla were identified within the bacterial 16S rRNA gene libraries in all soil samples. The top ten bacterial phyla (average relative abundance > 1%) were *Proteobacteria* (22.68–34.21%), *Actinobacteria* (14.04–23.42%), *Acidobacteria* (11.00–16.43%), *Bacteroidetes* (6.67–8.63%), *Chloroflexi* (4.90–6.95%), *Gemmatimonadetes* (3.20–6.78%), *Firmicutes* (2.40–6.35%), *Verrucomicrobia* (2.65–3.42%), *Planctomycetes* (2.28–3.06%), and *Cyanobacteria* (0.35–1.89%) (Figure 4A), accounting for more than 90.57% of the detectable reads in all soil samples. The four land-use types varied in their relative abundance of the top ten bacterial phyla (Figure 4A). The relative abundance of *Actinobacteria* was lower in FL than in the other three land-use types (*p* < 0.05). SL had significantly increased relative abundance of *Acidobacteria* and *Gemmatimonadetes* and decreased relative abundance of *Proteobacteria* compared with ML (*p* < 0.05, Figure 4A). The relative abundance of other taxa also varied significantly among land-use types. For example, BL exhibited the lowest relative abundance of *Firmicutes* (*p* < 0.05). However, no significant difference was found for the relative abundance of *Bacteroidetes*, *Cyanobacteria*, *Verrucomicrobia*, and *Planctomycetes* among the four land-use types (Figure 4B).

Fifteen phyla were identified within the fungal ITS gene libraries in all soil samples. The top five fungal phyla (average relative abundance > 1%) were *Ascomycota* (38.18–56.71%), *Mortierellomycota* (1.77–38.37%), *Basidiomycota* (3.39–11.75%), *Mucoromycota* (0.09–2.34%), and *Chytridiomycota* (0.12–1.56%) (Figure 4B), accounting for more than 70.16% of the detectable reads in all soil samples. Fungi differed from bacteria in their responses to land-use types. *Mortierellomycota* was the only fungal phylum that had significantly different relative abundances between SL and ML soils (Figure 4B). In addition, *Blastocladiomycota* was not detected in BL, and *Zoopagomycota* was not detected in SL (Figure 4B). *Ascomycota* was the most abundant fungal group (38.18–56.71% relative abundance), but its relative abundance exhibited no significant differences among the four land-use types. *Mortierellomycota* was the second most abundant fungi phylum (1.77–38.37% relative abundance); its relative abundance was significantly increased in SL compared with ML (Figure 4B, *p* < 0.01). The third most abundant phylum was *Basidiomycota* (3.39–11.75% relative abundance), with no significant differences among the four land-use types (Figure 4B). Overall, these results revealed that fungal communities were less sensitive than bacterial communities to land-use changes. 

### 3.4. Soil Microbial Community Composition and Diversity

**Figure 5:**
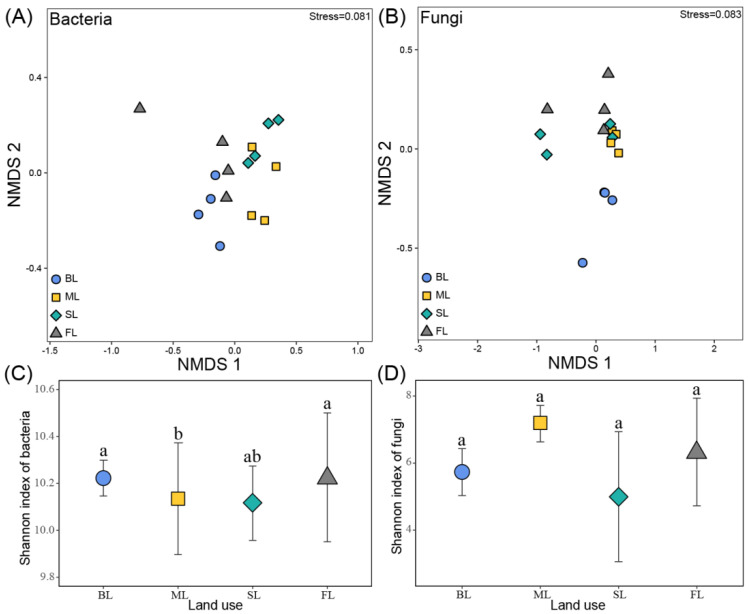
NMDS (**A**,**B**) and boxplot of Shannon diversity index (**C**,**D**) of bacterial and fungal communities in different land-use types. Different letters above boxplots indicate significant differences among land-use types (*p* ˂ 0.05) based on one-way ANOVA with the LSD test. Abbreviations: FL, forest land; SL, sugarcane land; ML, maize land; BL, barren land.

NMDS revealed that soil bacterial (R^2^ = 0.400, *p* < 0.001) and fungal (R^2^ = 0.305, *p* < 0.05) community composition differed significantly among land-use types (Figure 5). Analysis of similarities of soil bacterial community composition (Table 1) showed significant differences in all pairs of land-use types except for SL vs. ML. However, only the fungal community composition in ML was different than that in the other three land-use types (Table 1, Figure 5B). Lower values of Shannon indices were detected for bacteria in ML compared to SL and BL (*p* < 0.05, Figure 5C), while there were no significant differences in fungal diversity across land-use types (Figure 5D). Both methods and analyses showed that fungal communities were less affected than bacterial communities by land-use changes.

### 3.5. Relationships among Soil Microbial Community Abundance and Soil Physicochemical Properties

**Figure 6:**
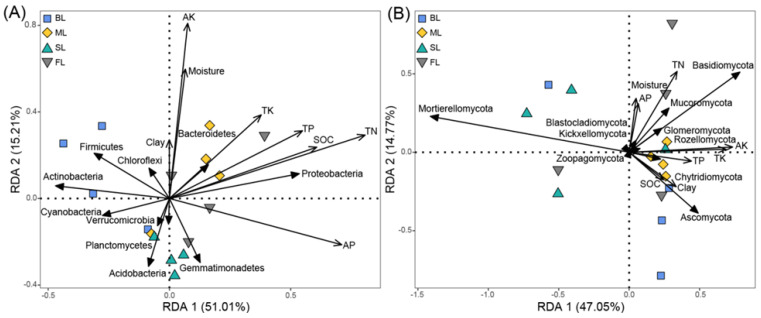
Redundancy analysis (RDA) biplot of bacterial (**A**) and fungal (**B**) community composition in different land-use types constrained by soil physicochemical properties. Abbreviations: FL, forest land; SL, sugarcane land; ML, maize land; BL, barren land; TN, soil total nitrogen; TP, soil total phosphorus; TK, soil total potassium; SOC, soil organic carbon; AK, available potassium; AP, available phosphorus.

**Figure 7:**
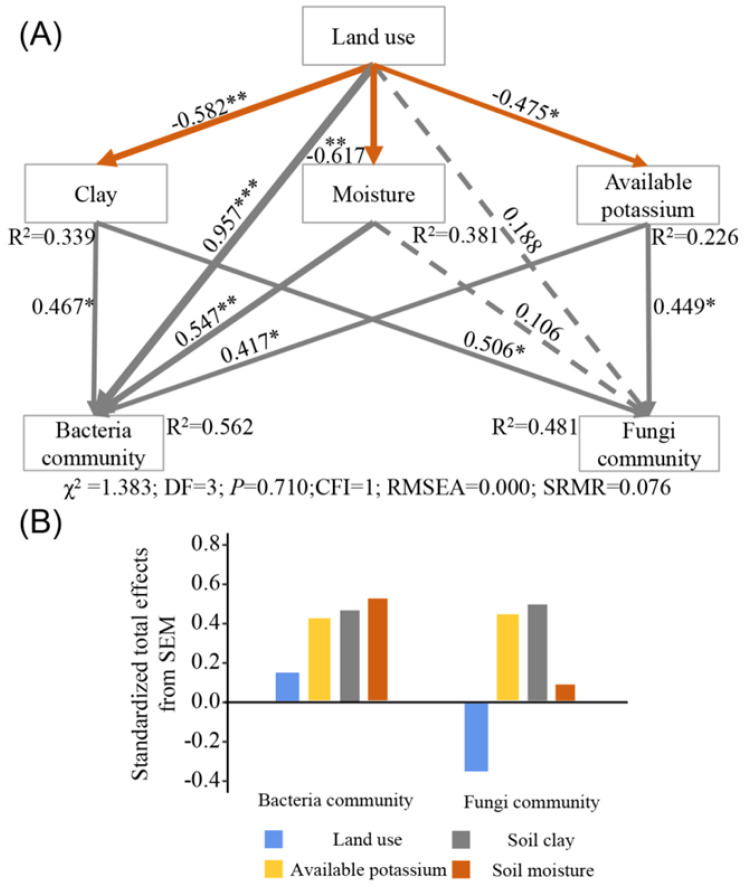
Structural equation model (SEM, **A**) showing the direct and indirect effects of land-use and soil physicochemical properties on the relative abundance of soil bacterial and fungal phyla (**B**). Note: Continuous arrows and dashed arrows indicate significant and non-significant relationships, respectively. The significance level is represented by * (*p* < 0.05), ** (*p* < 0.01), *** (*p* < 0.001). Numbers adjacent to arrows indicate actual *p*-values; width of the arrow is proportional to the size of the path coefficient. The gray and orange arrows indicate positive and negative relationships, respectively.

The results of RDA showed that the first and second axes explained 51.01% and 15.21% (Figure 6A) and 47.05% and 14.77% (Figure 6B) of total variation in bacterial and fungal community composition, respectively. The relative abundance of bacterial phyla was positively correlated with TN, SOC, and AK contents (Table 2), and these variables accounted for 33.3, 15.1, and 10.2% of the variation in bacterial community composition. The relative abundance of fungal phyla was positively correlated with AK, which explained 22.1% of the variation in fungal community composition (Table 2). SEM described 56.2 and 48.1% of the variance in bacterial and fungal community composition, respectively (Figure 7A). Land-use type had direct negative effects on soil clay, soil moisture, and AK contents. In addition, land-use indirectly affected the relative abundance of bacterial and fungal phyla through effects on soil physicochemical properties. Specifically, soil clay, soil moisture, and AK contents were strong drivers of the relative abundance of bacterial phyla, which were 46.7, 54.7, and 41.7% of the variance. The variability in the relative abundance of fungal phyla was primarily driven by soil clay and AK contents, which explained 50.6 and 44.9% of the variance. However, the effect of soil moisture on the relative abundance of fungal phyla was not significant. The effect of land-use on the relative abundance of bacterial and fungal phyla exhibited opposite trends, as indicated by the standardized total effects from SEM (Figure 7B). It is worth noting that land-use, per se, significantly affected the relative abundance of bacterial but not of fungal phyla (Figure 7A).

## 4. Discussion

Consistent with our hypothesis, we observed that SL decreased SOC and TN contents compared to maize cultivation and conversion of forest land to farmland (Figure 2). These differences could be due to the harvesting practices for maize and sugarcane. In sugarcane fields, nearly all of the aboveground biomass was harvested and removed each year, so that aboveground organic matter inputs were minimal. In maize fields, stubbles were retained after harvest, which could benefit the accumulation of SOC and TN. Another possible explanation could be a difference in nutrient utilization [49]. Sugarcane prefers to absorb potassium [50,51,52]. We observed that SL had the lowest soil moisture and potassium content. Similarly, the negative effect of sugarcane planting on clay content was observed. The significant decrease in clay content in SL may be related to whole-stem harvesting of sugarcane and its soil preparation by hoe prior to planting [30,53]. Overall, our results suggest that long-term sugarcane monoculture may result in a decrease in soil carbon, nitrogen, and potassium pools, thereby preventing the sustainability of this production system.

Our results showed that land-use changes could result in variability in the abundance of different soil bacteria and fungi at the phylum level (Figure 4). We observed that *Proteobacteria* was higher in FL, ML, and SL than in BL. *Proteobacteria* were prevalent in farmland and forest land [54] and could decompose and utilize litter fall [34]. The lowest relative abundance of *Proteobacteria* in BL may be due to the absence of litter input. In contrast, the lowest relative abundance of *Actinobacteria* was in FL, where SOC was the highest. *Actinobacteria* decreased mineralization rates in some soils, which enhanced SOC accumulation [55,56,57]. Moreover, as *Acidobacteria* prefer low-nutrient soils, their presence indicates soil degradation [58,59]. In this study, the relative abundance of *Acidobacteria* was higher in SL than in other land uses, indicating that sugarcane planting could result in soil degradation. In addition, some taxonomic groups were affected by certain land-use types, leading to changes in community composition. For example, among the top ten abundant fungi at the phylum level, *Blastocladiomycota* was absent in barren land, whereas *Zoopagomycota* was absent in sugarcane fields. Furthermore, both bacterial and fungal diversity was the lowest in sugarcane fields. Thus, sugarcane monoculture had negative impacts on soil health, evidenced by modifications in specific microbial taxa after two years of land-use changes. Similar results were reported by Kwong et al. (2002) and Medina et al. (2013), where whole-plant harvesting and low litter return resulted in low-nutrient soil in sugarcane plots and a low abundance of microorganisms [23,60,61].

Additionally, community composition, diversity, and the dominant bacteria phyla changed with land-use, whereas fungi were less affected (Figure 5 and Figure 6). This indicated that the soil bacterial community in the Dry-Hot Valley was more sensitive to land-use changes than the fungal community. These results are consistent with those of other studies in grasslands and forestlands. Pesaro et al. (2004) found that the bacterial community was readily influenced by soil conditions in a silt loam grassland, while the fungal community exhibited insignificant changes [62]. Xu et al. (2021) also found that the response of the bacterial community to variability in SOC in grassland and forestland was more sensitive than that of fungi [63]. The differences in responses between bacteria and fungi are most likely due to the utilization of different resources. Bacteria utilize soluble organic carbon and nitrogen compounds with simple molecular structures, such as monosaccharides and disaccharides [64], whereas fungi are more likely to utilize complex polysaccharides, such as lignin and cellulose [65,66]. Usually, soil carbon, nitrogen, and soluble nutrients are the first elements to vary after land-use changes [67], and this may cause a rapid response in soil bacterial communities. Furthermore, our SEM analysis showed that changes in soil moisture caused by land-use exerted a significant positive effect on the relative abundance of bacterial but not fungal phyla (Figure 7). One possible explanation is that fungal hyphae facilitate access to soil moisture [68], and the chitinous cell walls can enhance resistance to drying [69]. Moreover, changes in the clay content caused by land-use had a significant effect on bacterial and fungal community structures. Higher clay content can improve soil connectedness, which can promote the utilization of available nutrients by bacteria and fungi [70].

Potassium is mainly derived from the weathering of rocks [71]. Unlike soil carbon and nitrogen, potassium cannot be directly used by microorganisms as growth factors [66]. In this study, the RDA and SEM results revealed that land-use affected the relative abundance of bacterial and fungal phyla via the AK content, indicating that AK content is an important indicator of variability in microbial phyla in the Dry-Hot Valley. Potassium is an essential element that regulates intracellular pH in vivo and facilitates microbial uptake of soil nutrients by regulating cell osmotic pressure [72]. Several studies have reported that fungi can rapidly take up potassium and that the diversity of fungal communities is strongly associated with potassium content [72,73]. In addition, soil bacteria affects the solubility and availability of potassium, which conversely affects the selection of specific bacteria related to potassium concentration [16,74,75]. However, further studies are needed to understand the potential mechanism of the influence of AK on bacterial and fungal community composition in different land uses.

## 5. Conclusions

This study demonstrated that sugarcane land and barren land decreased soil organic carbon and total nitrogen contents. Changes in soil moisture, clay, and potassium contents due to land-use were key indicators of variability in microbial composition. Moreover, bacterial communities (including diversity) were remarkably more sensitive to land-use than fungi. Although land-use changes in the Dry-Hot Valley region are extensive, selection of new crops for cultivation requires careful assessment because tropical cash crops such as sugarcane hinder sustainability. Furthermore, maintenance of soil functions depends on the tight linkages between aboveground plants and belowground microbiota. Therefore, changes in abiotic factors of soil clay and moisture as well as biotic factors of the specific bacterial phyla can potentially reverse soil degradation caused by changes in land-use. We propose that applications of potassium fertilizer and judicious irrigation in the Dry-Hot Valley may mitigate the negative effects of unsustainable land-use, particularly that of sugarcane cultivation.

## Figures and Tables

**Table 1:** Analysis of similarities among soil bacterial (left) and fungal (right) community composition in four land uses based on 999 permutations.

Bacteria			Fungi		
Vs_Group	R	*p*	Vs_Group	*R* ^1^	*p*
SL-ML	0.17	0.17	SL-ML	0.36	0.03
SL-BL	0.86	0.04	SL-BL	0.39	0.18
SL-FL	0.49	0.03	SL-FL	0.15	0.15
ML-BL	0.85	0.02	ML-BL	0.53	0.03
ML-FL	0.42	0.03	ML-FL	0.40	0.03
BL-FL	0.47	0.02	BL-FL	0.40	0.06

^1^*R* is the proportion of variance explained, and *p* < 0.05 was considered significant.

**Table 2:** Results of the summarized effects of explanatory variables.

Bacteria				Fungi			
Soil Property	Explains (%)	pseudo-F	*p*	Name	Explains (%)	pseudo-F	*p*
TN	33.3	7.0	0.002 *	AK	22.1	4.0	0.020 *
SOC	15.1	3.8	0.012 *	SOC	11.6	2.3	0.078
AK	10.2	3.0	0.030 *	TK	6.6	1.4	0.244
TK	6.9	2.2	0.082	Moisture	5.8	1.2	0.364
TP	3.0	0.9	0.447	AP	5.7	1.0	0.400
Moisture	2.3	0.7	0.536	Clay	5.2	1.1	0.384
Clay	2.4	0.7	0.528	TP	4.8	0.9	0.458
AP	1.3	0.3	0.746	TN	4.4	0.9	0.430

* *p* < 0.05.

## Data Availability

The datasets obtained in this study can be found in online repositories. Raw sequencing datasets for this study can be accessed from the NCBI Sequence Read Archive (SRA) Database: https://catalog.data.gov/dataset/sequence-read-archive-sra, (Accessed on 11 July 2021); BioProject number of bacteria is PRJNA742498 and of fungi is PRJNA742518. The soil physicochemical properties of the four land uses can be found in the Supplementary Material (Supplementary Table S1).

## Supplementary Materials

The following are available online at https://www.mdpi.com/article/10.3390/microorganisms10050956/s1, Table S1. Soil physicochemical properties in four land-use types. Figure S1. Rarefaction curves of 16S rRNA (A) and ITS (B) sequencing data in four land-use types.

## References

[1] Fedele G., Locatelli B., Djoudi H., Colloff M.J. (2018). Reducing risks by transforming landscapes: Cross-scale effects of land-use changes on ecosystem services. PLoS ONE.

[2] Schjønning P., Jensen J.L., Bruun S., Jensen L.S., Christensen B.T., Munkholm L.J. (2018). The Role of Soil Organic Matter for Maintaining Crop Yields: Evidence for a Renewed Conceptual Basis. Adv. Agron..

[3] Song X., Hansen M.C., Stehman S.V., Potapov P.V., Tyukavina A., Vermote E.F., Townshend J.R. (2018). Global land change from 1982 to 2016. Nature.

[4] Guo L.B., Gifford R.M. (2002). Soil carbon stocks and land use change: A meta-analysis. Glob. Chang. Biol..

[5] Dong Y., Xiong D., Su Z., Li J., Yang D., Shi L., Liu G. (2014). The distribution of and factors influencing the vegetation in a gully in the Dry-hot Valley of southwest China. Catena.

[6] Wang G., Huang W., Zhou G., Mayes M.A., Zhou J. (2020). Modeling the processes of soil moisture in regulating microbial and carbon-nitrogen cycling. J. Hydrol..

[7] Borrelli P., Robinson D.A., Panagos P., Lugatod E., Yangb J.Y., Alewell C., Wuepper D., Montanarella L., Ballabio C. (2020). Land use and climate change impacts on global soil erosion by water (2015—2070). Proc. Natl. Acad. Sci. USA.

[8] Mcdougall R., Rader R., Kristiansen P. (2020). Urban agriculture could provide 15% of food supply to Sydney, Australia, under expanded land use scenarios. Land Use Policy.

[9] Li B., Liu Y., Zhang H., Liu Y., Liu Y., Xie P. (2021). Research progress in the functionalization of microcystin-LR based on interdisciplinary technologies. Coord. Chem. Rev..

[10] Roy K., Ghosh D., DeBruyn J.M., Dasgupta T., Wommack K.E., Liang X., Wagner R.E., Radosevich M. (2020). Temporal Dynamics of Soil Virus and Bacterial Populations in Agricultural and Early Plant Successional Soils. Front. Microbiol..

[11] Six J., Paustian K. (2014). Aggregate-associated soil organic matter as an ecosystem property and a measurement tool. Soil Biol. Biochem..

[12] Yang X., Leng Y., Zhou Z., Shang H., Ni K., Ma L., Yi X., Cai Y., Ji L., Ruan J. (2022). Ecological management model for the improvement of soil fertility through the regulation of rare microbial taxa in tea (*Camellia sinensis* L.) plantation soils. J. Environ. Manag..

[13] Ramírez P.B., Fuentes-Alburquenque S., Díez B., Vargas I., Bonilla C.A. (2020). Soil microbial community responses to labile organic carbon fractions in relation to soil type and land use along a climate gradient. Soil Biol. Biochem..

[14] Ji L., Yang Y., Yang L., Zhang D. (2020). Effect of land uses on soil microbial community structures among different soil depths in northeastern China. Eur. J. Soil Biol..

[15] Li Y., Xie X., Zhu Z., Liu K., Liu W., Wang J. (2022). Land use driven change in soil organic carbon affects soil microbial community assembly in the riparian of Three Gorges Reservoir Region. Appl. Soil Ecol..

[16] Emmert E.A.B., Geleta S.B., Rose C.M., Seho-Ahiable G.E., Hawkins A.E., Baker K.T., Evans A.S., Harris M.E., Mrozinski A.C., Folkoff M.E. (2021). Effect of land use changes on soil microbial enzymatic activity and soil microbial community composition on Maryland’s Eastern Shore. Appl. Soil Ecol..

[17] Xu A., Liu J., Guo Z., Wang C., Pan K., Zhang F., Pan X. (2021). Soil microbial community composition but not diversity is affected by land-use types in the agro-pastoral ecotone undergoing frequent conversions between cropland and grassland. Geoderma.

[18] Mhete M., Eze P.N., Rahube T.O., Akinyemi F.O. (2020). Soil properties influence bacterial abundance and diversity under different land-use regimes in semi-arid environments. Sci. Am..

[19] Gömöryová E., Barančíková G., Tobiašová E., Halás J., Skalský R., Koco Š., Gömöry D. (2020). Responses of soil microorganisms to land use in different soil types along the soil profiles. Soil Water Res..

[20] Naldini M.B., Pérez Harguindeguy N., Kowaljow E. (2021). Soil carbon release enhanced by increased litter input in a degraded semi-arid forest soil. J. Arid. Environ..

[21] Chernov T.I., Zhelezova A.D., Tkhakakhova A.K., Ksenofontova N.A., Zverev A.O., Tiunov A.V. (2021). Soil microbiome, organic matter content and microbial abundance in forest and forest-derived land cover in Cat Tien National Park (Vietnam). Appl. Soil Ecol..

[22] Medorio-García H.P., Alarcón E., Flores-Esteves N., Montaño N.M., Perroni Y. (2020). Soil carbon, nitrogen and phosphorus dynamics in sugarcane plantations converted from tropical dry forest. Appl. Soil Ecol..

[23] de Vries F.T., Hoffland E., van Eekeren N., Brussaard L., Bloem J. (2006). Fungal/bacterial ratios in grasslands with contrasting nitrogen management. Soil Biol. Biochem..

[24] Rousk J., Brookes P.C., Bååth E. (2010). Investigating the mechanisms for the opposing pH relationships of fungal and bacterial growth in soil. Soil Biol. Biochem..

[25] Fanin N., Bertrand I. (2016). Aboveground litter quality is a better predictor than belowground microbial communities when estimating carbon mineralization along a land-use gradient. Soil Biol. Biochem..

[26] Veldkamp E., Schmidt M., Powers J.S., Corre M.D. (2020). Deforestation and reforestation impacts on soils in the tropics. Nat. Rev. Earth Environ..

[27] Zhang R.Z. (1992). Dry Valley in Hengduan Mountainous Region.

[28] Xiong D., Zhou H., Yang Z., Zhang X. (2005). Slope lithologic property, soil moisture condition and revegetation in dry-hot valley of jinsha river. Chin. Geogr. Sci..

[29] Long H., Zhang D., He G. (2017). The effects of planted and plowed *Stylosanthes guianensis* on degrading soil fertility in hilly countries of dry-hot valley. Acta Ecol. Sin..

[30] Rong L., Duan X., Zhang G., Gu Z., Feng D. (2019). Impacts of tillage practices on ephemeral gully erosion in a dry-hot valley region in southwestern China. Soil Tillage Res..

[31] Li Z., Sun X., Huang Z., Zhang X., Wang Z., Li S., Zheng W., Zhai B. (2020). Changes in nutrient balance, environmental effects, and green development after returning farmland to forests: A case study in Ningxia, China. Sci. Total Environ..

[32] Li S., Huang X., Lang X., Shen J., Xu F., Su J. (2020). Cumulative effects of multiple biodiversity attributes and abiotic factors on ecosystem multifunctionality in the Jinsha River valley of southwestern China. For. Ecol. Manag..

[33] Li Y., Duan X., Li Y., Li Y., Zhang L. (2021). Interactive effects of land use and soil erosion on soil organic carbon in the dry-hot valley region of southern China. Catena.

[34] Yang D., Xiong D., Zhang B., Guo M., Su Z., Dong Y., Zhang S., Xiao L., Lu X. (2017). Effect of grass basal diameter on hydraulic properties and sediment yield processes in gully beds in the dry-hot valley region of Southwest China. Catena.

[35] Yuan Y., Xiong D., Wu H., Zhang S., Zhang B., Dahal N.M., Liu L., Li W., Zhang W., Shi L. (2020). Spatial variation of soil physical properties and its relationship with plant biomass in degraded slopes in dry-hot valley region of Southwest China. J. Soil Sediments.

[36] FAO (2006). World Reference Base for Soil Resources.

[37] Duka I., Shallari S., Maçi A., Shehu J. (2015). Heavy Metal Concentration and Physico-Chemical Parameters in Koder Kamza Soils. J. Agric. Sci..

[38] Song J., Duan X., Han X., Li Y., Li Y., He D. (2019). The accumulation and redistribution of heavy metals in the water-level fluctuation zone of the Nuozhadu Reservoir, Upper Mekong. Catena.

[39] Nelson D.W., Sommers L.E. (1982). Total carbon, organic carbon and organic matter. Methods of Soil Analysis Part 2, Chemical and Microbial Properties.

[40] Liu G.S. (1996). Soil Physics and Chemistry Analysis and Description of Soil Profiles.

[41] Bao S.D. (2000). Soil Agri-Chemistrical Analysis.

[42] Mori H., Maruyama F., Kato H., Toyoda A., Dozono A., Ohtsubo Y., Nagata Y., Fujiyama A., Tsuda M., Kurokawa K. (2014). Design and Experimental Application of a Novel Non-Degenerate Universal Primer Set that Amplifies Prokaryotic 16S rRNA Genes with a Low Possibility to Amplify Eukaryotic rRNA Genes. DNA Res..

[43] White T., Bruns T., Lee S., Taylor J., Gelfand D., Sninsky J., White T. (1990). Amplification and Direct Sequencing of Fungal Ribosomal RNA Genes for Phylogenetics.

[44] Ward T., Larson J., Meulemans J., Hillmann B., Lynch J., Sidiropoulos D., Spear J.R., Gaporaso G., Blekhman R., Knight R. (2017). BugBase predicts organism level microbiome phenotypes. BioRxiv.

[45] Rognes T., Flouri T., Nichols B., Quince C., Mahé F. (2016). VSEARCH: A versatile open source tool for metagenomics. PeerJ.

[46] Haas B.J., Gevers D., Earl A.M., Feldgarden M., Ward D.V., Giannoukos G., Ciulla D., Tabbaa D., Highlander S.K., Sodergren E. (2011). Chimeric 16S rRNA sequence formation and detection in Sanger and 454-pyrosequenced PCR amplicons. Genome Res..

[47] Edgar R.C. (2013). UPARSE: Highly accurate OTU sequences from microbial amplicon reads. Nat. Methods.

[48] Lozupone C., Knight R. (2005). UniFrac: A new phylogenetic method for comparing microbial communities. Appl. Environ. Microbiol..

[49] Delgado-Baquerizo M., Maestre F.T., Reich P.B., Jeffries T.C., Gaitan J.J., Encinar D., Berdugo M., Campbell C.D., Singh B.K. (2016). Microbial diversity drives multifunctionality in terrestrial ecosystems. Nat. Commun..

[50] Wu J., Liu W., Chen C. (2016). Can intercropping with the world’s three major beverage plants help improve the water use of rubber trees?. J. Appl. Ecol..

[51] Watanabe K., Yamamoto T., Yamada T., Sakuratani T., Nawata E., Noichana C., Sributta A., Higuchi H. (2004). Changes in seasonal evapotranspiration, soil water content, and crop coefficients in sugarcane, cassava, and maize fields in Northeast Thailand. Agr. Water Manag..

[52] Estrada-Bonilla G.A., Durrer A., Cardoso E.J.B.N. (2021). Use of compost and phosphate-solubilizing bacteria affect sugarcane mineral nutrition, phosphorus availability, and the soil bacterial community. Appl. Soil Ecol..

[53] Cherubin M.R., Franco A.L.C., Guimarães R.M.L., Tormena C.A., Cerri C.E.P., Karlen D.L., Cerri C.C. (2017). Assessing soil structural quality under Brazilian sugarcane expansion areas using Visual Evaluation of Soil Structure (VESS). Soil Tillage Res..

[54] Li D., Liu J., Chen H., Zheng L., Wang K. (2018). Soil microbial community responses to forage grass cultivation in degraded karst soils, Southwest China. Land Degrad. Dev..

[55] Lewin G.R., Carlos C., Chevrette M.G., Horn H.A., McDonald B.R., Stankey R.J., Fox B.G., Currie C.R. (2016). Evolution and Ecology of Actinobacteria and Their Bioenergy Applications. Annu. Rev. Microbiol..

[56] Rodrigues A.A., Araujo M.V.F., Soares M.D.S., Oliveira B.F.R.D., Sibov S.T., Vieira J.D.G. (2018). Isolation and Screening for Multi-trait Plant Growth Promoting Actinobacteria From Organic Sugarcane Rhizosphere. Int. J. Microbiol. Res..

[57] Praeg N., Seeber J., Leitinger G., Tasser E., Newesely C., Tappeiner U., Illmer P. (2020). The role of land management and elevation in shaping soil microbial communities: Insights from the Central European Alps. Soil Biol. Biochem..

[58] Ward N.L., Challacombe J.F., Janssen P.H., Henrissat B., Coutinho P.M., Wu M., Xie G., Haft D.H., Sait M., Badger J. (2009). Three Genomes from the PhylumAcidobacteria Provide Insight into the Lifestyles of These Microorganisms in Soils. Appl. Environ. Microbiol..

[59] Manpoong C., De Mandal S., Bangaruswamy D.K., Perumal R.C., Benny J., Beena P.S., Ghosh A., Kumar N.S., Tripathi S.K. (2020). Linking rhizosphere soil biochemical and microbial community characteristics across different land use systems in mountainous region in Northeast India. Meta Gene.

[60] Xun W., Huang T., Zhao J., Ran W., Wang B., Shen Q., Zhang R. (2015). Environmental conditions rather than microbial inoculum composition determine the bacterial composition, microbial biomass and enzymatic activity of reconstructed soil microbial communities. Soil Biol. Biochem..

[61] Knappová J., Pánková H., Münzbergová Z. (2016). Roles of Arbuscular Mycorrhizal Fungi and Soil Abiotic Conditions in the Establishment of a Dry Grassland Community. PLoS ONE.

[62] Pesaro M., Nicollier G., Zeyer J., Widmeret F. (2004). Impact of soil drying-rewetting stress on microbial communities and activities and on degradation of two crop protection products. Appl. Environ. Microbiol..

[63] Xu H., Du H., Zeng F., Song T., Peng W. (2021). Diminished rhizosphere and bulk soil microbial abundance and diversity across succession stages in Karst area, southwest China. Appl. Soil Ecol..

[64] Boer W.D., Folman L.B., Summerbell R.C., Boddy L. (2005). Living in a fungal world: Impact of fungi on soil bacterial niche development. FEMS Microbiol. Rev..

[65] Ewbank G., Edwards D., Abbott G.D. (1996). Chemical characterization of Lower Devonian vascular plants. Org. Geochem..

[66] Tardy V., Spor A., Mathieu O., Lévèque J., Terrat S., Plassart P., Regnier T., Bardgett R.D., van der Putten W.H., Roggero P.P. (2015). Shifts in microbial diversity through land use intensity as drivers of carbon mineralization in soil. Soil Biol. Biochem..

[67] Haghverdi K., Kooch Y. (2020). Soil carbon and nitrogen fractions in response to land use/cover changes. Acta Oecol..

[68] Orwin K.H., Dickie I.A., Wood J.R., Bonner K.I., Holdaway R.J. (2015). Soil microbial community structure explains the resistance of respiration to a dry–rewet cycle, but not soil functioning under static conditions. Funct. Ecol..

[69] Preece C., Verbruggen E., Liu L., Weedon J.T., Peñuelas J. (2019). Effects of past and current drought on the composition and diversity of soil microbial communities. Soil Biol. Biochem..

[70] Wang G., Or D. (2013). Hydration dynamics promote bacterial coexistence on rough surfaces. ISME J..

[71] Brady N.C., Weil R.R. (2017). The Nature and Properties of Soils.

[72] Pan X., Zhang S., Zhong Q., Gong G., Wang G., Guo X., Xu X. (2020). Effects of soil chemical properties and fractions of Pb, Cd, and Zn on bacterial and fungal communities. Sci. Total Environ..

[73] Weed S.B., Davey C.B., Cook M.G. (1969). Weathering of Mica by Fungi. Soil Sci. Soc. Am. J..

[74] Miransari M. (2013). Soil microbes and the availability of soil nutrients. Acta Physiol. Plant.

[75] Pereira L.B., Vicentini R., Ottoboni L.M.M., Moustafa A. (2014). Changes in the bacterial community of soil from a neutral mine drainage channel. PLoS ONE.

