# Supplementary material for: Land-Use Driven Changes in Soil Microbial Community Composition and Soil Fertility in the Dry-Hot Valley Region of Southwestern China
Source: Microorganisms. 2022 May 2;10(5):956. doi: 10.3390/microorganisms10050956 (PMC9146041; doi:10.3390/microorganisms10050956)
Supplement: Supplementary file 1 [file microorganisms-10-00956-s001.zip › microorganisms-1682595-supplementary.pdf]

*microorganisms*

*Supplementary Materials*

**Table S1.** Soil physicochemical properties in four land-use types.

| Land use | SOC $(mg.g^{-1})$ | TN $(mg.g^{-1})$ | TP $(mg.g^{-1})$ | AK $(mg.g^{-1})$ | AP $(mg.g^{-1})$ | Moisture (%) | Clay (%) | Sand (%) | pH |
|---|---|---|---|---|---|---|---|---|---|
| BL1 | 18.95 | 1.44 | 0.2582 | 0.2625 | 0.0230 | 20.86 | 39.70 | 46.41 | 7.43 |
| BL2 | 21.98 | 1.79 | 0.2722 | 0.2304 | 0.0189 | 16.90 | 37.47 | 42.36 | 7.42 |
| BL3 | 20.92 | 1.62 | 0.2348 | 0.2349 | 0.0237 | 21.54 | 39.18 | 33.88 | 7.39 |
| BL4 | 19.27 | 1.72 | 0.2990 | 0.2775 | 0.0157 | 19.85 | 40.70 | 40.00 | 7.26 |
| ML1 | 34.60 | 2.35 | 0.3047 | 0.2867 | 0.0593 | 15.44 | 39.59 | 36.24 | 7.60 |
| ML2 | 43.83 | 2.34 | 0.4117 | 0.3483 | 0.0600 | 18.75 | 30.11 | 36.95 | 7.23 |
| ML3 | 26.58 | 2.24 | 0.3025 | 0.2503 | 0.0585 | 14.90 | 40.17 | 34.53 | 6.82 |
| ML4 | 31.11 | 2.37 | 0.2920 | 0.2808 | 0.0634 | 15.53 | 42.76 | 34.36 | 6.90 |
| SL1 | 22.21 | 2.06 | 0.2613 | 0.1378 | 0.0437 | 17.02 | 30.82 | 38.30 | 6.86 |
| SL2 | 20.97 | 1.75 | 0.2758 | 0.1376 | 0.0518 | 16.05 | 28.35 | 38.30 | 6.50 |
| SL3 | 24.61 | 1.73 | 0.2546 | 0.1852 | 0.0474 | 14.80 | 30.71 | 36.54 | 7.36 |
| SL4 | 23.83 | 1.87 | 0.2778 | 0.1898 | 0.0589 | 16.02 | 30.70 | 36.54 | 7.12 |
| FL1 | 31.97 | 3.11 | 0.2865 | 0.2575 | 0.0338 | 24.90 | 42.94 | 28.01 | 7.46 |
| FL2 | 32.12 | 2.30 | 0.2865 | 0.2483 | 0.0559 | 19.58 | 42.06 | 26.18 | 7.47 |
| FL3 | 25.29 | 3.60 | 0.2932 | 0.2287 | 0.0441 | 23.69 | 40.76 | 25.41 | 7.33 |
| FL4 | 22.99 | 3.87 | 0.2799 | 0.2939 | 0.0346 | 23.94 | 40.12 | 23.77 | 7.34 |

Abbreviations: FL, land conversion to forest; SL, sugarcane land; ML, maize land; BL, barren land; SOC, soil organic carbon; TN, soil total nitrogen; TP, soil total phosphorus; AK, available potassium; AP, available phosphorus.

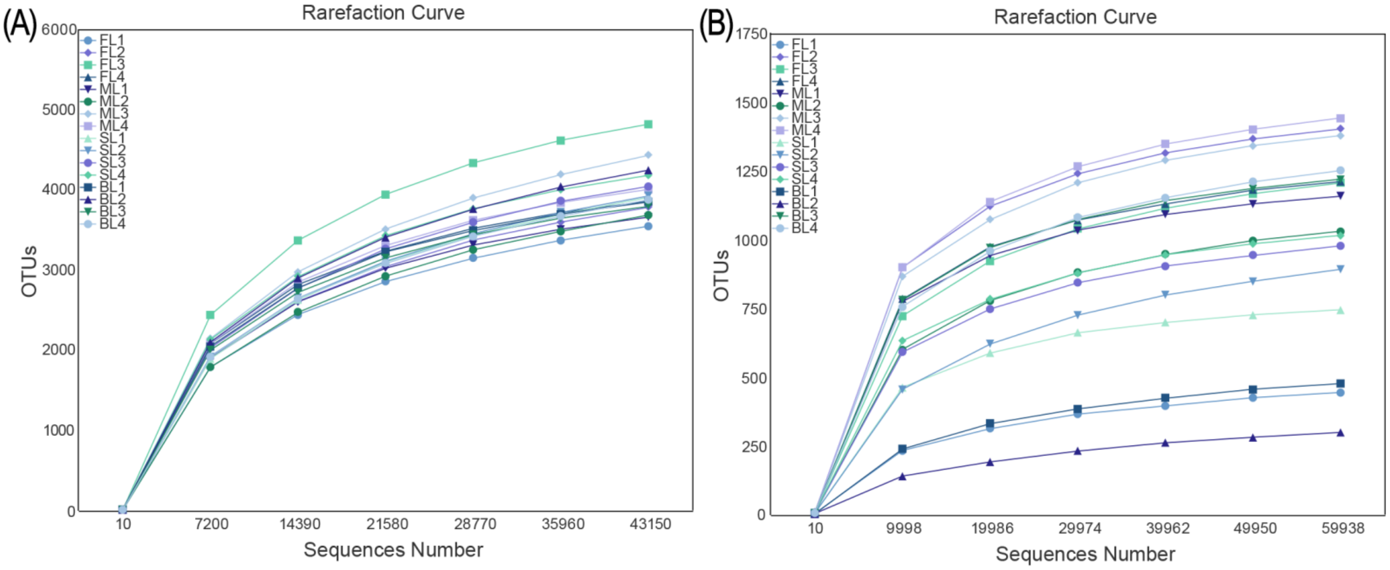


**Figure S1.** Rarefaction curves of 16S rRNA (**A**) and ITS (**B**) sequencing data in four land-use types. Abbreviations: FL, land conversion to forest; SL, sugarcane land; ML, maize land; BL, barren land. Note: Each colour represents an individual sample.
